# Association of maternal KIR gene content polymorphisms with reduction in perinatal transmission of HIV-1

**DOI:** 10.1371/journal.pone.0191733

**Published:** 2018-01-23

**Authors:** Yusuf O. Omosun, Anna J. Blackstock, John Williamson, Anne Maria van Eijk, John Ayisi, Juliana Otieno, Renu B. Lal, Feiko O. ter Kuile, Laurence Slutsker, Ya Ping Shi

**Affiliations:** 1 Division of Parasitic Diseases and Malaria, Center for Global Health, Centers for Disease Control and Prevention, Atlanta, Georgia, United States of America; 2 Atlanta Research and Education Foundation, Atlanta, Georgia, United States of America; 3 Center for Global Health Research, Kenyan Medical Research Institute, Kisumu, Kenya; 4 Child and Reproductive Health Group, Liverpool School of Tropical Medicine, Liverpool, United Kingdom; 5 New Nyanza Provincial General Hospital, Ministry of Health, Kisumu, Kenya; 6 Division of Global HIV/AIDS, Center for Global Health, Centers for Disease Control and Prevention, Atlanta, Georgia, United States of America; University of Liverpool Institute of Infection and Global Health, UNITED KINGDOM

## Abstract

The role of killer cell immunoglobulin-like receptors (KIRs) in the transmission of HIV-1 has not been extensively studied. Here, we investigated the association of KIR gene content polymorphisms with perinatal HIV-1 transmission. The KIR gene family comprising 16 genes was genotyped in 313 HIV-1 positive Kenyan mothers paired with their infants. Gene content polymorphisms were presented as presence of individual KIR genes, haplotypes, genotypes and KIR gene concordance. The genetic data were analyzed for associations with perinatal transmission of HIV. There was no association of infant KIR genes with perinatal HIV-1 transmission. After adjustment for gravidity, viral load, and CD4 cell count, there was evidence of an association between reduction in perinatal HIV-1 transmission and the maternal individual KIR genes KIR2DL2 (adjusted OR = 0.50; 95% CI: 0.24–1.02, P = 0.06), KIR2DL5 (adjusted OR = 0.47; 95% CI: 0.23–0.95, P = 0.04) and KIR2DS5 (adjusted OR = 0.39; 95% CI: 0.18–0.80, P = 0.01). Furthermore, these maternal KIR genes were only significantly associated with reduction in perinatal HIV transmission in women with CD4 cell count ≥ 350 cells/ μl and viral load <10000 copies/ml. Concordance analysis showed that when both mother and child had KIR2DS2, there was less likelihood of perinatal HIV-1 transmission (adjusted OR = 0.44; 95% CI: 0.20–0.96, P = 0.039). In conclusion, the maternal KIR genes KIR2DL2, KIR2DL5, KIR2DS5, and KIR2DS2 were associated with reduction of HIV-1 transmission from mother to child. Furthermore, maternal immune status is an important factor in the association of KIR with perinatal HIV transmission.

## Introduction

Sub-Saharan Africa has a high number of people living with HIV/AIDS, with an estimated 2.3 million children living with HIV and about 190,000 children with new infections in 2014 [1]. Perinatal HIV transmission is the most common route of HIV infection in children. Perinatal transmission occurs during pregnancy, labor and delivery, or breastfeeding and is influenced by maternal viral load and immune status, the timing and route of delivery, viral subtype, and host genetic factors [2]. Studies have shown significant reduction in the mother-to-child transmission (MTCT) of HIV-1 by the use of highly active antiretroviral treatment in pregnant women or peripartum short-course antiretroviral therapy or single dose of antiviral drugs given at labor and delivery [3, 4]. However, in most resource-constrained areas of sub-Saharan Africa, the rate of MTCT remains high due to inaccessibility to antiretroviral drugs and the lack of adherence to antiretroviral drugs during pregnancy and the postpartum period [5–7].

The human killer immunoglobulin-like receptor (KIR) family of genes is part of the leukocyte receptor gene complex on chromosome 19 and comprises 16 individual KIR genes [8]. KIRs are expressed on the surface of NK cells and form a major part of the response mechanism of NK cells to external stimuli [9]. The KIR repertoire determines the functionality of NK cells and HLA class I molecules are ligands for KIRs [10–12]. The nomenclature of KIR is dependent on the number of extracellular immunoglobulin (Ig) domains (2D and 3D) and the length of their cytoplasmic tails (‘S’ for short or ‘L’ for long) [13]. The KIRs with short tails activate NK cell functions, while those with the long tails play an inhibitory role. The repertoire of NK cell KIRs is very different between individuals because of the diversity in the number of genes present (defined as gene content) and the number of alleles, as well as the difference in binding to HLA class I ligands [14]. In addition, at the population level, there is a higher diversity of KIR genes in sub-Saharan African compared to European populations, and this diversity could be partially shaped by higher birth rates and defense from various pathogens [12, 15].

Genetic association studies involving KIR with diseases have been concerned predominantly with viral infections, autoimmune diseases and malignancies [16]. Many of the previous investigations of KIR or KIR-HLA association with HIV have been focused mainly on disease progression in adults already infected with HIV [17–19], with few studies reporting the association of KIR or KIR-HLA with HIV transmission [20]. For instance, KIR3DS1 has been associated with higher NK cell effector functions in early HIV-1 disease, which may lead to better clinical outcomes [21, 22]. Studies on MTCT of HIV have been focused on the HLA ligand polymorphisms [23–25], with few assessing the associations between KIR polymorphisms and MTCT of HIV [26, 27]. Two studies were conducted in Johannesburg, South Africa, an area with no reported incidence of malaria [28]. The first study reported that KIR2DL2, KIR2DL3 and HLA-C ligands in mothers or infants were associated with reduction or increase in MTCT of HIV-1 respectively [26]. The second study reported that differences in the MTCT of HIV could be related to functional or nonfunctional variants of KIR2DS4 [27]. In another study in the US, perinatally HIV-1 infected children were found to have cytotoxically dysfunctional NK cells relative to perinatally exposed uninfected children, with these NK cells having increased KIR2DL3 expression which correlated with decreased CD4 T cell percentages [29].

The presence of *P*. *falciparum* in pregnant women increases HIV-1 viral load [30]. But the effect of malaria infection during pregnancy on perinatal HIV transmission is not clear, with several studies reporting conflicting conclusions, stating that malaria infection increased perinatal HIV-1 transmission [31, 32], had no effect on transmission [33], or had a protective effect [34]. Given the observed association between KIR gene polymorphisms and MTCT reported in non-malaria endemic area in South Africa [26, 27] and a possible interaction between MTCT and malaria infection in pregnant women, we conducted a pilot study to investigate the relationship between MTCT and KIR gene content polymorphisms in mother-child pairs from western Kenya where co-infection of HIV with malaria is very common.

## Materials and methods

### Study subjects and clinical procedures

The present study used samples from mothers and their infants from an epidemiological investigation of the relationship between placental malaria (PM) and perinatal transmission of HIV-1 [vertical transmission (VT study)] that was carried out in western Kenya between 1996 and 2001 [32, 35]. The Luo is the dominant ethnic group in the study area. During the VT study period, the prevalence of infections in pregnant women attending antenatal clinic was about 25% for HIV-1 and 20% for *P*. *falciparum*, respectively [36]. In the VT project, women were enrolled if they had singleton uncomplicated pregnancies of at least 32 weeks gestation and no known underlying chronic illnesses [32]. Information on reproductive history, socio-demographics, heath/clinical status, and malaria treatment was collected at enrollment and delivery. Blood samples were collected from mothers at enrollment, delivery, and one month postpartum for HIV diagnosis, HIV viral load, CD4 count, malaria diagnosis, and hemoglobin level determination. In addition, infants were followed up and blood samples collected monthly were used for HIV diagnosis. In the VT epidemiological study, HIV-positive women were given enrollment priority; while among HIV-negative women, women with PM were given enrollment priority. In total, 269 HIV-negative and 829 HIV-positive pregnant women were originally enrolled in the VT epidemiological study. For this host genetics study, we analyzed blood samples from 313 HIV-positive mothers paired with their infants for KIR gene content polymorphisms, based on availability of mother-infant paired samples.

### Ethical information

Counseling was provided to all women before and after HIV testing. At the time of the VT study (1996–2001), the Kenyan Ministry of Health recommended breastfeeding regardless of HIV status, and access to zidovudine or nevirapine was by then neither recommended by the Kenyan MOH nor available [37]. Written informed consent for participation in the study was obtained from the mothers for themselves and their infants. Study methods of the VT project, including the host genetics tests and analysis described here, were approved by the Kenya Medical Research Institute Ethical Review Committee, Nairobi, Kenya and the Institutional Review Board of the Centers for Disease Control and Prevention, Atlanta, USA.

### Laboratory procedures

#### HIV-related tests

Maternal HIV status was determined based on a combination of initial testing with Sero Strip HIV-1/2 (Saliva Diagnostic Systems, New York, USA) and confirmation with Capillus HIV-1/HIV-2 test (Cambridge Diagnostics, Cambridge, UK). Infant HIV status was monitored monthly by DNA polymerase chain reaction (PCR) using gpM-Z primers. Maternal CD4 cell count was determined using fluorescent-activated cell sorting analysis (FACScan, Becton Dickinson, San Jose, California, USA) based on manufacturer instructions. Maternal HIV-1 viral load at delivery was measured using the Roche Amplicor HIV-1 monitor test versions 1.0 and 1.5, respectively (Roche Molecular Systems, Branchburg, New Jersey, USA)[32].

#### Malaria-related tests

Thick smears made from placental and peripheral blood of mothers were stained with Giemsa and examined by microscopy. The number of asexual parasites/300 leukocytes was counted. Parasite density was estimated assuming 8000 leukocytes/μl. Peripheral blood hemoglobin concentrations (g/dl) were quantified using the HemoCue system (HemoCue, Brea, California) [32].

#### KIR Genotyping

The KIR genotyping method used in this study has been described previously [38]. Briefly, DNA was extracted from blood samples from 313 mother-infant pairs using the QIAamp DNA blood mini kit (Qiagen, Valencia, California, USA). KIR genotyping for 16 KIR genes was carried out using KIRSSO genotyping test (One Lambda Inc., Canoga Park, California, USA) based on the manufacturer instructions. The results were read on a Luminex 200 IS (Luminex Corp., Austin, Texas, USA). The presence of individual KIR genes was determined using HLA Fusion Beta software (One Lambda Inc., Canoga Park, California, USA). Positive control DNA samples with different profiles of KIR gene content from the International Histocompatibility Working Group (IHWG) were used in all experiments.

### Definitions

#### Clinical definitions

Infants were considered to be perinatally infected with HIV if they had two or more consecutive HIV-positive PCR tests, with the first positive PCR at or before 4 months of age[32]. Mothers of perinatally infected infants were classified as “transmitters” and those of uninfected infants as “non-transmitters.” Mothers of infants who acquired HIV at or after 5 months of age (considered postnatally acquired HIV) were also included in the analysis as non-transmitters [32]. Placental malaria was categorized into low (1–9999 parasites/μl) or high (≥ 10 000 parasites/μl) density per the parallel VT epidemiological study [32]. CD4 cell count was grouped as ≤ 200, 200–499, and ≥ 500cells/μl and viral load as <1000, 1000–9999 and ≥ 10000 copies/ml. Gravidity was divided into primi- or secundigradvida versus multigravida to allow assessment of possible differences in immunological environment between early and later pregnancies [35, 39].

#### Genetic definitions

KIR gene content polymorphisms were assessed in various ways: (1) the presence of 16 individual KIR genes [40]; (2) KIR haplotype A (presence of KIR3DL3, KIR2DL3, KIR2DL1, KIR2DP1, KIR3DP1, KIR2DL4, KIR3DL1, KIR2DS4 and KIR3DL2 only) and haplotype B (presence of KIR2DL1, KIR2DL2, KIR2DL4, KIR2DL5, KIR2DS1, KIR2DS2, KIR2DS3, KIR2DS5, KIR2DS5, KIR2DP1, KIR3DL2, KIR3DL3, KIR3DS1 and KIR3DP1) [41–43]; and (3) KIR genotypes AA, AB and BB. Genotype AA includes individuals with KIR3DL3, KIR2DL3, KIR2DL1, KIR2DP1, KIR3DP1, KIR2DL4, KIR3DL1, KIR2DS4, and KIR3DL2 while genotype BB comprises individuals without KIR2DL1, KIR2DL3, KIR3DL1 and KIR2DS4 [38]. Individuals not classified as either genotype AA or BB were regarded as genotype AB [44]. KIR concordance was defined as mothers and their infants having the same KIR gene content for single genes, haplotypes or genotypes.

### Statistical analysis

Statistical analyses compared HIV-transmitting mothers to those who did not transmit HIV in terms of characteristics of the mothers, characteristics of the babies, and KIR gene content of both mothers and babies.

To determine whether characteristics of mothers or babies affected HIV transmission in unadjusted analyses, exact Chi-square tests were used for categorical characteristics, and exact Wilcoxon tests were used for other characteristics. For all other comparisons, logistic regression models were used. Due to sparse data issues, the Firth likelihood penalty was used for logistic regression models where possible [45]. If the model using Firth penalty did not converge, exact logistic regression was used.

Both univariable and multivariable models were used to assess the relationship between HIV transmission status and KIR gene content of mothers or infants, where KIR gene content included single genes, genotype and haplotype. Based on the number of HIV transmitters, it was determined that multivariable models should control for no more than three variables to reduce the possibility of over fitting the data. Gravidity (<3 vs. ≥3), viral load (<10,000 copies/ml vs. ≥10,000 copies/ml), and CD4 count (<350 cells/ul vs. ≥350 cells /ul) were controlled for in the multivariable models as these were known predictors of MTCT of HIV [5, 46, 47].

Concordance models assessed whether a mother and child having the same single genes, haplotype, or genotype, affected HIV transmission. Again, both univariable and multivariable models were fit, and multivariable models controlled for gravidity, CD4 count, and viral load, which were categorized as mentioned above.

For several single genes that appeared related to HIV transmission from mother to child, models with interaction terms were used to assess whether the relationship between KIR gene content of mothers and MTCT differed for different levels of CD4 count or viral load, where CD4 count and viral load were dichotomized as mentioned above. Models controlled for gravidity and were fit for CD4 count and viral load separately.

The false discovery rate (FDR) was used to determine significance, controlling for multiple comparisons within each of the comparison groups separately, where comparison groups were defined by gene content type (single genes or not), whether the genes considered were from infants, mothers, or concordance between the two, and if the models used were adjusted. Two additional groups were defined for the interaction models, one group with interaction with CD4 and the other group with viral load interaction. Since there are two gene content types, three subject types, and two model types, plus two additional interaction model groups, there were 14 comparison groups for which FDR was used to determine significance individually.

## Results

### Characteristics of HIV transmission groups

HIV transmitters (n = 53) were more likely to have high maternal HIV-1 viral load and low maternal CD4 cell count than non-transmitters (n = 260) (Table 1), which is consistent with the findings from the previous epidemiological evaluation of VT study [32]. Also, transmitters were more likely to be primi- or secundigravidae and to have had an episiotomy or perineal tear compared to non-transmitter group (Table 1). There were no significant differences in maternal age, route of delivery, duration of labor, preterm-delivery, baby birth weight, maternal anemia, and placental malaria between HIV transmitters and non-transmitters (Table 1).

**Table 1:** Characteristics of perinatal HIV-1 transmission groups.

	HIV non-Transmitter %	HIV Transmitter %	P^a^ value
Number (N)	260	53	
Mothers age (mean years± SD)	22.7±4.5	22.0±4.0	0.34
Primi or secundigravidae pregnancies	59.6	75.5	**0.031**
Vaginal delivery	100	100	—
Duration of labor^b^ (hours)			
<10	39.6	30.8	0.27
Median time (IQR)	11.8 (7.8–18.0)	12.7 (8.8–19.2)	0.44
Episiotomy or perineal tear present	34.6	49.1	**0.061**
Preterm delivery^c^ [48]	6.5	13.2	0.15
Baby birth weight <2500 g^d^	3.5	7.6	0.25
Maternal anemia at delivery^e^ (<11 g/dl)	81.3	90.9	0.13
Placental malaria (parasites/μl)			
0	76.2	79.3	0.093
1–9999	20.0	11.3	
≥10,000	3.9	9.4	
Presence (any)	23.9	20.8	0.72
Maternal CD4 cell count^f^ (μl)			
<200	1.7	12.5	**<0.001**
200–499	33.3	41.7	
≥500	65.0	45.8	
Median (IQR)	584.5 (430.0–807.5)	463 (273.5–634.0)	**0.002**
Maternal viral load^g^ (copies/ml)			
<1000	54.2	25.5	**<0.001**
1000–9999	31.9	29.8	
≥10,000	14.0	44.7	
Median (IQR)	764.0 (200.0–4021.0)	7489.0 (707.0–34420.0)	**<0.001**

IQR, interquartile range. Note:

^a^ P-values are based on exact Pearson chi-square test for categorical variables and exact Wilcoxon test for others.

^b^ n = 312,

^c^ newborns were classified as preterm if delivery occurred before 37 weeks of gestation using a modified Dubowitz Method,

^d^ weight measured to the nearest gram on an electric balance within 24 h of birth,

^e^ n = 263,

^f^ n = 288,

^g^ n = 276.

### Effect of KIR gene content polymorphisms on perinatal transmission of HIV

First, we determined whether any individual KIR gene was associated with transmission status. Gravidity, viral load, and CD4 count were controlled for in the multivariable models as these were known predictors of MTCT of HIV. Overall frequencies of the KIR genes were similar between mothers and their infants. For infants, there was no association of individual KIR genes with transmission status. However, mothers that had KIR2DL2 (adjusted OR = 0.50, 95% CI = 0.24–1.02, P = 0.056), KIR2DL5 (adjusted OR = 0.47, 95% CI = 0.23–0.95, P = 0.035) and KIR2DS5 (adjusted OR = 0.39, 95% CI = 0.18–0.80, P = 0.010) delivered infants with decreased odds of being infected with HIV-1 (Table 2). However, after controlling for multiple comparisons the significant association of these genes with HIV-1 transmission was not observed. Overall frequencies of haplotypes and genotypes were similar between mothers and their infants. There was no association of infant and mother KIR haplotypes and genotypes with the odds of perinatal transmission of HIV-1 (Table 3).

**Table 2:** Relationship of individual KIR genes with perinatal transmission of HIV-1.

	HIV Non- Transmitter % N = 260	HIV Transmitter % N = 53	Unadjusted Odds ratio (95% CI^a^)	P value	Adjusted Odds ratio^b^ (95% CI^a^)	P value
Individual KIR Genes						
Infant						
KIR2DL1	98.1	96.2	0.44 (0.10–2.52)	0.32	0.47 (0.10–2.80)	0.37
KIR2DL2	48.9	47.2	0.94 (0.52–1.69)	0.83	0.66 (0.32–1.32)	0.24
KIR2DL3	85.8	86.8	1.04 (0.47–2.60)	0.93	0.91 (0.38–2.46)	0.85
KIR2DL4	99.6	100.0	0.62 (0.03–90.76)	0.78	0.07 (>0.002)	1.00
KIR2DL5	56.2	47.2	0.70 (0.39–1.26)	0.23	0.53 (0.26–1.06)	0.074
KIR2DP1	98.1	100.0	2.30 (0.26–304.06)	0.53	2.22 (0.17–316.69)	0.59
KIR2DS1	18.9	17.0	0.91 (0.40–1.89)	0.81	0.71 (0.27–1.68)	0.45
KIR2DS2	41.5	43.4	1.08 (0.60–1.95)	0.79	0.62 (0.29–1.29)	0.21
KIR2DS3	17.7	13.2	0.74 (0.30–1.63)	0.48	0.78 (0.28–1.90)	0.59
KIR2DS4	95.8	96.2	0.95 (0.27–5.00)	0.94	1.43 (0.35–8.47)	0.65
KIR2DS5	41.5	35.9	0.79 (0.43–1.45)	0.46	0.54 (0.25–1.12)	0.10
KIR3DL1	98.5	100.0	1.88 (0.20–250.15)	0.65	1.89 (0.14–274.73)	0.68
KIR3DP1	99.6	100.0	0.62 (0.03–90.76)	0.78	0.07 (>0.002)	1.00
KIR3DS1	10.8	11.3	1.12 (0.42–2.62)	0.81	1.21 (0.41–3.15)	0.72
Mother						
KIR2DL1	96.5	100.0	4.04 (0.50–523.98)	0.24	2.20 (0.23–297.03)	0.57
KIR2DL2	54.2	35.9	0.48 (0.26–0.87)	0.015*	0.50 (0.24–1.02)	0.056*
KIR2DL3	85.0	88.7	1.30 (0.57–3.45)	0.55	1.54 (0.58–4.87)	0.41
KIR2DL4	99.2	100.0	1.04 (0.08–143.52)	0.98	0.54 (0.04–76.40)	0.72
KIR2DL5	58.9	39.6	0.46 (0.25–0.84)	0.011*	0.47 (0.23–0.95)	0.035*
KIR2DP1	98.9	100.0	1.46 (0.14–196.64)	0.80	0.54 (0.04–76.40)	0.72
KIR2DS1	22.7	9.4	0.38 (0.14–0.90)	0.026*	0.46 (0.15–1.16)	0.10
KIR2DS2	47.7	34.0	0.57 (0.31–1.04)	0.07	0.62 (0.29–1.25)	0.18
KIR2DS3	18.1	15.1	0.84 (0.36–1.79)	0.66	1.01 (0.39–2.35)	0.99
KIR2DS4	96.9	98.1	1.18 (0.26–11.23)	0.85	1.96 (0.22–257.23)	0.62
KIR2DS5	48.1	28.3	0.44 (0.22–0.81)	0.008*	0.39 (0.18–0.80)	0.010*
KIR3DL1	98.9	100.0	1.46 (0.14–196.64)	0.80	0.67 (0.06–92.40)	0.80
KIR3DP1	99.2	100.0	1.04 (0.08–143.52)	0.98	0.54 (0.04–76.40)	0.72
KIR3DS1	10.8	9.4	0.93 (0.32–2.27)	0.87	0.99 (0.32–2.63)	0.99

Note: Logistic regression with Firth likelihood penalty was used for all models except KIR2DL4 and KIR3DP1 adjusted models for infants, which were fit using exact logistic regression due to convergence issues. For these two models, the estimate reported is a median unbiased estimate (MUE).

^a^CI = Confidence interval.

^b^Adjusted odds ratios derived from multivariable logistic regression controlling for CD4 cell count, viral load, and gravidity. False Discovery Rate (FDR) correction was used to determine significance.

‘*’ Signifies uncorrected P-value that is < 0.05 but is non-significant after correction.

**Table 3:** Relationship of KIR haplotypes and genotypes with perinatal transmission of HIV-1.

	HIV Non- Transmitter % N = 260	HIV Transmitter % N = 53	Unadjusted Odds ratio (95% CI^a^)	P value	Adjusted Odds ratio^b^ (95% CI^a^)	P value
**Infant**						
*KIR Haplotype*						
Haplotype A	37.8	35.9	0.93 (0.50–1.70)	0.82	1.29 (0.63–2.62)	0.48
Haplotype B^c^	62.3	64.2	1	—	1	—
*KIR Genotype*						
AA	37.3	34.0	0.86 (0.46–1.58)	0.64	1.15 (0.56–2.33)	0.70
AB^c^	61.9	66.0	1	—		—
BB	0.8	0.0	0.91 (0.01–11.50)	0.95	0.62 (0.004–11.75)	0.77
**Mother**						
*KIR Haplotype*						
Haplotype A	33.5	47.2	1.77(0.98–3.21)	0.060	1.74 (0.86–3.70)	0.14
Haplotype B^c^	66.5	52.8	1	—	1	—
*KIR Genotype*						
AA	32.7	47.2	1.82 (1.00–3.29)	0.050	1.79 (0.86–3.70)	0.12
AB^c^	66.5	52.8	1	—	1	—
BB	0.8	0.0	1.22(0.01–15.48)	0.90	2.35 (0.02–33.11)	0.62

Note: Logistic regression with Firth likelihood penalty was used for all models.

^a^CI = Confidence interval.

^b^Adjusted odds ratios derived from multivariable logistic regression controlling for CD4 cell count, viral load, and gravidity.

^c^Reference group was selected based on the group with highest frequency. False Discovery Rate (FDR) correction was used to determine significance.

### Effect of KIR gene concordance on perinatal transmission of HIV-1

Because KIR gene family is highly polymorphic, we evaluated the association of KIR concordance for single genes, haplotypes and, genotypes on the odds of MTCT of HIV-1. Gravidity, viral load, and CD4 count were controlled for in the multivariable models as these were known predictors of MTCT of HIV. Our results show that only KIR2DS2 (adjusted OR = 0.44, 95% CI = 0.20–0.96, P = 0.04) when present in both mother and child was associated with a lowered odds of HIV-1 perinatal infection (Table 4), however, after controlling for multiple comparisons the significant association of KIR2DS2 concordance with HIV-1 transmission was not observed. In addition, there was no association of KIR2DS2 alone in mother and child with viral load (data not shown).

**Table 4:** Relationship of individual KIR gene concordance with perinatal transmission of HIV-1.

	%Concordance^a^,HIV Non- Transmitters (N = 260)	%Concordance^a^,HIV Transmitters (N = 53)	Unadjusted Odds ratio (95% CI^b^)	P value	Adjusted Odds ratio^c^ (95% CI^b^)	P value
***Individual KIR Genes***						
KIR2DL1	96.2	96.2	0.86 (0.24–4.58)	0.84	0.66 (0.16–3.77)	0.60
KIR2DL2	72.3	66.0	0.74 (0.40–1.40)	0.35	0.78 (0.36–1.72)	0.52
KIR2DL3	81.5	90.6	2.01 (0.85–5.73)	0.12	1.96 (0.74–6.51)	0.19
KIR2DL4	99.6	100.0	0.62 (0.03–90.76)	0.78	0.44 (0.02–64.42)	0.64
KIR2DL5	72.7	69.8	0.86 (0.46–1.66)	0.64	0.87 (0.39–2.01)	0.73
KIR2DP1	97.7	100.0	2.73 (0.32–358.40)	0.43	2.35 (0.19–331.06)	0.55
KIR2DS1	83.1	81.1	0.85 (0.42–1.88)	0.68	0.93 (0.39–2.39)	0.87
KIR2DS2	74.6	60.4	0.52 (0.28–0.96)	0.038*	0.44 (0.20–0.96)	0.039*
KIR2DS3	84.2	83.0	0.89 (0.42–2.02)	0.76	0.74 (0.31–1.88)	0.51
KIR2DS4	95.0	94.3	0.79 (0.26–3.14)	0.71	1.51 (0.37–8.86)	0.59
KIR2DS5	72.7	73.6	1.03 (0.54–2.04)	0.94	0.81 (0.38–1.82)	0.60
KIR3DL1	98.1	100.0	2.30 (0.26–304.06)	0.53	2.14 (0.18–302.89)	0.60
KIR3DP1	99.6	100.0	0.62 (0.03–90.76)	0.78	0.44 (0.02–64.42)	0.64
KIR3DS1	89.2	86.8	0.76 (0.34–1.93)	0.54	0.62 (0.25–1.68)	0.33

Note: Logistic regression with Firth likelihood penalty was used for all models.

^a^ Concordance is defined as mother and her child having the same type of KIR gene present.

^b^CI = Confidence interval.

^c^Adjusted odds ratios derived from multivariable logistic regression controlling for CD4 cell count, viral load, and gravidity. False Discovery Rate (FDR) correction was used to determine significance.

‘*’ Signifies uncorrected P-value that is < 0.05 but is non-significant after correction.

### Effect of CD4 count and HIV viral load on the association between KIR gene content polymorphisms and perinatal transmission of HIV-1

As reported above, maternal KIR2DL2, KIR2DL5 and KIR2DS5 were in part associated with reduction in MTCT of HIV-1 (Table 2). To understand the influence of the immune and disease status of the mothers, we further assessed effect of CD4 cell count and viral load on the associations between the observed maternal KIR gene content polymorphisms and the odds of perinatal transmission of HIV-1. Models were fit for CD4 cell count and viral load separately, and all models controlled for gravidity.

We categorized viral load into two groups (HIV viral load <10,000 copies/ml and HIV viral load ≥10,000 copies/ml) in this analysis. KIR2DL2, KIR2DL5 and KIR2DS5 were significantly associated with decreased odds of perinatal transmission of HIV-1 only in the women with viral loads less than 10,000 copies/ml (Table 5). CD4 cell count was categorized into two groups (CD4 cell count <350 cells/μl and CD4 cell count ≥350 cells/μl). KIR2DL5 and KIR2DS5 were significantly associated with decreased odds of perinatal transmission of HIV-1 only in the women with CD4 cell count greater than 350 cells/μl (Table 5). After FDR correction for multiple comparisons the significant association of KIR2DL5 and KIR2DS5 with perinatal HIV-1 transmission in lower viral load group remained significant.

**Table 5:** Effect of CD4 cell count and HIV viral load on the relationship of maternal KIR2DL2, KIR2DL5, & KIR2DS5 with perinatal transmission of HIV-1.

Gene	Parameter	% Transmitted HIV		Adjusted OR(95%CI)	P value
		Gene Present	Gene Absent	Odds ratio^b^ (95% CI^a^)	
KIR2DL2	CD4 cell count, cells/ml				
	<350	28.57 (8/28)	42.86 (9/21)	0.52 (0.16–1.69)	0.28
	≥350	9.40 (11/117)	16.39 (20/122) (20/122) (20/122)	0.53 (0.24–1.12)	0.097
	HIV-1 load, copies/ml				
	<10,000	6.84 (8/117)	16.98 (18/106)	0.37 (0.15–0.85)	0.019*
	≥10,000	34.62 (9/26)	44.44 (12/27)	0.61 (0.20–1.83)	0.38
KIR2DL5	CD4 cell count, cells/ml				
	<350	27.59 (8/29)	45.00 (9/20)	0.47 (0.14–1.53)	0.21
	≥350	9.23 (12/130)	17.43 (19/109)	0.47 (0.21–1.00)	0.048*
	HIV-1 load, copies/ml				
	<10,000	5.56 (7/126)	19.59 (19/97)	0.25 (0.10–0.59)	0.001**
	≥10,000	41.94 (13/31)	36.36 (8/22)	1.11 (0.36–3.46)	0.86
KIR2DS5	CD4 cell count, cells/ml				
	<350	27.27 (6/22)	40.74 (11/27)	0.53 (0.15–1.72)	0.29
	≥350	7.55 (8/106)	17.29 (23/133)	0.38 (0.16–0.86)	0.018*
	HIV-1 load, copies/ml				
	<10,000	4.81 (5/104)	17.65 (21/119)	0.25 (0.09–0.64)	0.003**
	≥10,000	37.50 (9/24)	41.38 (12/29)	0.67 (0.21–2.04)	0.48

Note: Logistic regression with Firth likelihood penalty was used for all models. Analysis for this table includes all HIV positive women (n = 313). Due to missingness, CD4 cell count models have N = 288 (CD4 <350: n = 49; CD4 ≥ 350: n = 239) and HIV-1 load models have N = 276 (HIV-1 load <10,000: n = 223; HIV-1 load ≥ 10,000: n = 53).

^a^CI = Confidence interval.

^b^Adjusted odds ratios derived from multivariable logistic regression, controlling for gravidity. False Discovery Rate (FDR) correction was used to determine significance.

‘*’ Signifies uncorrected P-value that is < 0.05 but is non-significant after correction.

‘**’ Signifies uncorrected P-value that is significant after correction.

## Discussion

There is very little information regarding the effect of KIR gene polymorphisms on the MTCT of HIV. Furthermore, no information is available in areas where HIV and malaria co-infections are common. The objective of this study was to examine the relationship between KIR gene content polymorphisms and the perinatal transmission of HIV-1 in a malaria endemic area. Presence of KIR2DL2, KIR2DL5 and KIR2DS5 in the mothers, but not in infants, was associated with decreased odds of HIV infection in infants. Moreover, these maternal KIR genes were only significantly associated with reduction in perinatal transmission of HIV in the women with higher CD4 cell count and lower viral load. In addition, we found that when both mother and child had KIR2DS2, there was decreased odds of HIV being transmitted from mother to child.

In this study, presence of KIR2DL2, KIR2DL5 and KIR2DS5 in mothers, but not in infants, was associated with decreased odds of MTCT of HIV. It remains unclear what mechanisms are involved at the placental level that might result in this phenomenon. We speculate that interaction between maternal KIRs and fetal HLA ligands might play a role in determining maternal transmissibility of and infant susceptibility to HIV. This hypothesis needs to be tested. Nevertheless, the lower odds of MTCT among mothers with KIR2DL2 that was observed in this study is consistent with a previous study conducted in a non-malaria area in South Africa that showed that maternal inhibitory KIR2DL2 protected newborns from HIV infection [26]. There are more variants of KIR2DL5 and KIR2DS5 in people of African descent compared with other groups [49–51]. The participants in the study were of African ancestry; hence the correlation of KIR2DL5 and KIR2DS5, with reduction in MTCT of HIV might be unique to Africans living in a malaria endemic area.

In this study, the decreased odds of perinatal HIV transmission associated with KIR2DL2, KIR2DL5 or KIR2DS5 was observed only in the mothers with high CD4 count or low viral load seems predictable, presuming that immunocompromised status, indicated by low CD4 count or high viral load, causes high maternal transmissibility of the HIV virus. The observation also suggests that the association of the KIR genes with a reduction in MTCT of HIV might be secondary effect. One explanation for the results observed in this study could be that HIV positive women with high CD4 count maintain their general immune capacity including KIR regulatory role; as such, the undamaged KIR regulatory function in these women would then modulate the functionality of NK cells and lower the maternal transmissibility of HIV virus. There might also be a difference in expression of maternal HLA ligands that could depend on their CD4 count as well as the difference in expression of HLA ligands in the fetus. A previous study supports this speculation, showing differences in HLA class I allele expression between individuals with low and high CD4+ T cell count [52].

Concordance, which has been associated with disease transmission, occurs when mother and child have the same set of genes. This phenomenon has been studied in MTCT of HIV and most of the genes that have been studied are HLA genes [24]. Some studies noted that concordance in certain HLA alleles leads to increased risk of perinatal transmission [24, 53]. In this study, we showed that KIR2DS2 gene concordance reduced perinatal transmission of HIV-1. KIR2DS2 is almost in complete linkage disequilibrium (LD) with KIR2DL2 in the general population [54] and our previous study had shown that KIR2DS2 and KIR2DL2 were in significant LD in HIV-1 negative Kenyan pregnant women [38]. In addition, KIR2DS2 and KIR2DL2 are often co-expressed on NK cells [18, 55]. As discussed earlier, maternal KIR2DL2 is associated with lower odds of HIV transmission from mothers to their babies. Therefore, the association of KIR2DS2 concordance with lower odds of MTCT of HIV that was observed in this study might be due to an additive effect of KIR2DS2 on the KIR2DL2 in mothers, while KIR2DS2 alone in infants could play a role of reducing their susceptibility to HIV virus. Indeed, earlier immunological studies have shown that NK cells expressing KIR2DS2 on their surface are good responders to HIV peptides [56, 57].

Our pilot study has some limitations. First, because of limited blood sample volume we did not test the NK cells from the study participants to show their *in vitro* anti-HIV functions, and we also did not determine what subsets of KIR content were expressed on the NK cell surface. Second, we did not perform experiments to test for HLA ligand genes in this study mainly because of the focus on our initial study objective, which was to investigate the relationship between perinatal transmission of HIV-1 and KIR gene content polymorphisms. Third, the sample size for HIV-1 transmitter group was small, which might be limiting the levels of significance that we observed in this study, particularly after FDR correction. However, the results from this study have shown that the amount of information garnered from analyzing the KIR genes alone provides some interesting context, which could lead to confirmation of the results observed in this study in other populations and further investigation of KIR alleles, HLA ligands and functionality of NK cells in relation to MTCT with or without other co-infections.

## Conclusion

This study provides new insights on the potential importance of KIR genes in preventing MTCT of HIV-1 in malaria endemic areas. In this study, there were no associations between infant and mother KIR haplotypes and genotypes with MTCT, with only maternal KIR2DL2, KIR2DL5 and KIR2DS5 being associated with decreased odds of perinatal HIV transmission. Furthermore, these maternal KIR genes were only significantly associated with reduced odds of perinatal HIV transmission in the women with higher CD4 T-cell count and lower viral load, suggesting that maternal immune status could have influenced the association of KIR genes with a reduction in MTCT of HIV. In addition, this study showed that KIR2DS2 was associated with a decreased odds of HIV perinatal transmission when both mother and child have this gene, suggesting a possible additive effect of KIR2DS2 on KIR2DL2 in mothers but KIR2DS2 alone in the infants decreases the odds of MTCT of HIV. This study adds to the existing body of knowledge on the role of KIR genes in HIV transmission from mother to child. Further studies looking at the different KIR alleles as well as maternal and fetal HLA ligands in malaria endemic areas will be important.
